# Metabolomic biomarkers of the mediterranean diet in pregnant individuals: A prospective study

**DOI:** 10.1016/j.clnu.2023.01.011

**Published:** 2023-01-14

**Authors:** Liwei Chen, Jin Dai, Zhe Fei, Xinyue Liu, Yeyi Zhu, Mohammad L. Rahman, Ruijin Lu, Susanna D. Mitro, Jiaxi Yang, Stefanie N. Hinkle, Zhen Chen, Yiqing Song, Cuilin Zhang

**Affiliations:** a Department of Epidemiology, Fielding School of Public Health, University of California Los Angeles, Los Angeles, CA, 90095, USA; b Department of Biostatistics, Fielding School of Public Health, University of California Los Angeles, Los Angeles, CA, 90095, USA; c Division of Research, Kaiser Permanente Northern California, Oakland, CA, 94612, USA; d Division of Cancer Epidemiology and Genetics, National Cancer Institute, National Institutes of Health, Rockville, MD, USA; e Eunice Kennedy Shriver National Institute of Child Health and Human Development, National Institute of Health, Bethesda, MD, USA; f Global Center for Asian Women's Health, and Bia-Echo Asia Centre for Reproductive Longevity & Equality, Yong Loo Lin School of Medicine, National University of Singapore, 117549, Singapore; g Department of Obstetrics and Gynecology, Yong Loo Lin School of Medicine, National University of Singapore, 119228, Singapore; h Department of Biostatistics, Epidemiology, and Informatics, Perelman School of Medicine, University of Pennsylvania, Philadelphia, PA 19104, USA; i Department of Epidemiology, Indiana University Richard M. Fairbanks School of Public Health, Indianapolis, IN, USA

**Keywords:** Metabolomics, Pregnant individuals, Plasma biomarkers, Mediterranean diet, Dietary patterns

## Abstract

**Background and aims:**

Metabolomic profiling is a systematic approach to identifying biomarkers for dietary patterns. Yet, metabolomic markers for dietary patterns in pregnant individuals have not been investigated. The aim of this study was to identify plasma metabolomic markers and metabolite panels that are associated with the Mediterranean diet in pregnant individuals.

**Methods:**

This is a prospective study of 186 pregnant individuals who had both dietary intake and metabolomic profiles measured from the Fetal Growth Studies-Singletons cohort. Dietary intakes during the peri-conception/1st trimester and the second trimester were accessed at 8–13 and 16–22 weeks of gestation, respectively. Adherence to the Mediterranean diet was measured by the alternate Mediterranean Diet (aMED) score. Fasting plasma samples were collected at 16–22 weeks and untargeted metabolomics profiling was performed using the mass spectrometry-based platforms. Metabolites individually or jointly associated with aMED scores were identified using linear regression and least absolute shrinkage and selection operator (LASSO) regression models with adjustment for potential confounders, respectively.

**Results:**

Among 459 annotated metabolites, 64 and 41 were individually associated with the aMED scores of the diet during the peri-conception/1st trimester and during the second trimester, respectively. Fourteen metabolites were associated with the Mediterranean diet in both time windows. Most Mediterranean diet-related metabolites were lipids (e.g., acylcarnitine, cholesteryl esters (CEs), linoleic acid, long-chain triglycerides (TGs), and phosphatidylcholines (PCs), amino acids, and sugar alcohols. LASSO regressions also identified a 10 metabolite-panel that were jointly associated with aMED score of the diet during the peri-conception/1st trimester (AUC: 0.74; 95% CI: 0.57, 0.91) and a 3 metabolites-panel in the 2nd trimester (AUC: 0.68; 95% CI: 0.50, 0.86).

**Conclusion:**

We identified plasma metabolomic markers for the Mediterranean diet among pregnant individuals. Some of them have also been reported in previous studies among non-pregnant populations, whereas others are novel. The results from our study warrant replication in pregnant individuals by future studies.

**Clinical trial registration number:**

This study was registered at ClinicalTrials.gov.

## 1. Introduction

In the past two decades, growing evidence has consistently indicated the importance of dietary patterns as a measure of overall dietary quality, rather than individual nutrients or foods, in promoting health and preventing disease risk [1-3]. The Mediterranean diet, a traditional dietary pattern among people living in the Mediterranean Basin, featured higher intakes of vegetables, fruits, nuts, legumes, fish, cereals, and olive oil, but lower intakes of red and processed meats and sweets [4,5]. The favorable cardiometabolic and neurological effects of the Mediterranean diet have been demonstrated in both high-quality randomized controlled trials (RCT) [6] and systematic reviews and meta-analyses across different populations [7-9], including pregnant individuals [10,11]. Yet, molecular markers of the Mediterranean diet have not been elucidated in pregnant individuals.

Biomarkers of dietary intake can be applied as objective measures of dietary patterns and help to understand the underlying biological pathways between diet and health outcomes [12]. As compared to the traditional approach of examining biomarkers for a single nutrient or food separately (which likely overlooks the interactions among nutrients and food groups), the recent advance in high-throughput untargeted metabolomic profiling techniques permits a more comprehensive and systematic approach to identifying biomarkers for dietary patterns [13]. By measuring down-stream small molecules or metabolic products (<1.5 kDa, metabolomic markers), the metabolomics approach may provide more information on the interactions between nutrients/foods and genes for individuals and could identify novel biomarkers or biological pathways. Such an approach is ideal for identifying the complex, net impact of numerous nutrients and their metabolism in human bodies for a given dietary pattern.

Recent studies have investigated the metabolomic markers for the Mediterranean diet in non-pregnant populations, with each study having identified several blood metabolomic markers for the Mediterranean [14-23]. Yet, no studies have investigated the metabolomic markers for this healthy dietary pattern in pregnant individuals. Pregnancy can result in a series of dynamic physiological changes, including alterations of the maternal hormonal profile, basal metabolic rate, energy storage, and partition [24]. Thus, the metabolic responses to diet in pregnant individuals may not be the same as the non-pregnant populations. Furthermore, no previous studies have performed supervised methods to identify panels of dietary patterns related to metabolites. As dietary patterns are combinations of foods and nutrients, a panel of metabolites could better capture the multidimensionality and interrelations of nutrients and foods presented in the dietary patterns. Therefore, the primary aim of this study is to identify blood metabolomic markers and metabolite panels that are associated with the Mediterranean diet in pregnant individuals.

## 2. Methods

### 2.1. Study population and design

This was a prospective study among racially diverse pregnant individuals who enrolled in the *Eunice Kennedy Shriver* National Institute of Child Health and Human Development (NICHD) Fetal Growth Studies-Singletons cohort (FGS). Details of the cohort have been described previously [25]. Briefly, a total of 2802 individuals aged 18–40 years with singletons were recruited between 8 and 13 weeks of gestation from 12 clinical sites in the United States between July 2009 and January 2013. Institutional Review Board approval was obtained for all participating clinical sites, the data coordinating center, and NICHD. The current analysis only included 186 individuals from a nested gestational diabetes mellitus (GDM) case–control study within the FGS who had both blood plasma metabolomic profiling data and dietary intakes [26]. The participant flow chart is presented in Supplementary Fig. 1.

### 2.2. Assessment of dietary intakes and calculation of alternate mediterranean scores

We assessed individual's dietary intakes in the past 3 months (i.e., during the peri-conception and 1st trimester) at the baseline visit (8–13 weeks) using a semi-quantitative Food Frequency Questionnaire (FFQ) and during the second trimester (16–22 weeks) using the automated self-administered 24-h dietary recall (ASA24^®^; version Beta, 2011). Both dietary assessment tools were developed and validated by the National Cancer Institute, National Institutes of Health (NIH) [27-29]. We measured the adherence to the Mediterranean diet by calculating the alternate Mediterranean score (aMED) using the foods and nutrients data from the FFQ and ASA24. The aMED score was calculated from 8 food and nutrient components (i.e., fruits, vegetables, whole grains, nuts, legumes, fish, red and processed meats, and monounsaturated-to-saturated fat ratio), with the healthy components, scored 1 for above the median intake and 0 for below, and unhealthy components scored reversely [30]. Thus, a higher score indicated a better adherence to the Mediterranean diet. This method has been applied to calculate the alternate Mediterranean score in the same cohort in previous publications [10,11].

### 2.3. Biospecimen collection and metabolomics profiling and data pretreatment

We collected the fasting blood samples in the 2nd trimester (91.0% of the blood draw were within 16–22 weeks). Plasma samples were immediately processed and stored at −80.0 °C until analysis. We performed the untargeted metabolomic profiling at the NIH West Coast Metabolomics Center at the University of California Davis using the two platforms: high-throughput liquid chromatography-quadrupole time of flight mass spectrometry (LC-QTOF-MS/MS) and gas chromatography-time of flight mass spectrometry (GC-TOF-MS) [31]. All samples were analyzed by the two platforms. Internal standards were used for the calibration of retention times. A total of 751 features were detected, with 459 annotated metabolites and 292 unknown features. In the current study, we only included the 459 annotated metabolites. All 459 metabolites had missing values < 20% and missing values were imputed with half of the minimum value by batch, which is recommended for metabolomics data [32]. To correct day-to-day technical variation from the platform, metabolites were divided by their median values (i.e., re-scaled to a median of 1) and log-transformed within each batch [33].

Of the total 459 known metabolites, 353 were lipids or lipid-like molecules (76.9%), followed by 55 organic acids and derivatives (12.0%), 30 organic oxygen compounds (6.5%), 12 organo-heterocyclic compounds (2.6%), and 9 “others” (including 3 homogeneous non-metal compounds, 3 nucleosides, nucleotides and analogues, 2 benzenoids, and 1 organic nitrogen compound) (Supplementary Fig. 2).

### 2.4. Covariates

We collected individuals’ sociodemographic characteristics, lifestyle factors, and reproductive and medical history using a detailed questionnaire at study enrollment. Pre-pregnancy body mass index (BMI) was calculated based on height measured at baseline and self-reported pre-pregnancy weight. We categorized individuals into normal weight (19.0–24.9 kg/m^2^), overweight (25.0–29.9 kg/m^2^), or obese (≥30.0 kg/m^2^) by their pre-pregnancy BMI. We assessed physical activity using a validated Pregnancy Physical Activity Questionnaire (PPAQ) [34] in the first trimester for measuring habitual physical activity in the past 12 months and second trimester for physical activity since the baseline visit.

### 2.5. Statistical methods

We applied sampling weights in the statistical analyses to account for the oversampling of individuals with GDM in nested-case control samples and to represent the results in the full FGS sample. We described and compared individuals’ characteristics at the study enrollment across the aMED score tertiles. We presented the results as weighted percentages (%) and actual frequency (N) for categorical variables and weighted mean (standard errors, SE) for continuous variables. We calculated the P-values comparing individuals across aMED score tertiles by one-way Analysis of variance (ANOVA) tests for continuous variables and χ^2^-tests for categorical variables.

Several steps were performed to identify plasma metabolomic markers for aMED score. We used the aMED scores derived from the FFQs (assessed at baseline visit of 8–13 weeks) in the prospective analyses and the aMED scores derived from the ASA24^®^ (assessed at 16–22 weeks) during the 2nd trimester in the cross-sectional analyses. We first identified plasma metabolites that were individually associated with aMED score using the linear regressions with adjustment for potential confounders, including maternal age (years), race/ethnicity (non-Hispanic White, non-Hispanic Black, Hispanic, Asian/Pacific Islander), education (high-school degree or less, associate degree or more), pre-pregnancy BMI (kg/m^2^), and total physical activity (minutes/week). Metabolites were selected if they were significantly different, comparing the highest aMED tertile to the lowest tertile. We controlled for multiple comparisons by using the Benjamini-Hochberg method, with the overall false discovery rate (FDR) < 0.05 being considered statistically significant [35]. We also conducted the sensitivity analyses of the associations of individual metabolites with aMED score, stratified by the GDM status (i.e., within individuals who developed GDM and individuals who didn't develop GDM in the late 2nd semester). We performed the least absolute shrinkage and selection operator (LASSO) regressions to select the panel of plasma metabolites jointly associated (i.e., with) with aMED score [36]. The metabolites panels were selected if they had non-zero coefficients based on the criteria of lambda.1se. To avoid over-fitting, we performed the 10-fold cross-validation in LASSO regression with participants randomly divided into training and validation sets with a ratio of 2:1 and calculated the area under the curve (AUC) of each panel. All data analyses were conducted using SAS software (version 9.4; SAS Institute, Cary, NC, US) or R (version 4.0.2; R Studio: Integrated Development for R. R Studio, Inc., Boston, MA, US).

## 3. Results

### 3.1. Participants baseline characteristics

Among all individuals, 27.2% were non-Hispanic White, 25.7% were non-Hispanic Black, 24.4% were Hispanic, and 22.7% were Asian/Pacific Islander. The mean (SE) age of individuals at enrollment was 28.0 (0.4) years, and the mean BMI was 25.5 (0.4) kg/m^2^, with 50.4% having a pre-pregnancy BMI in the normal range. The baseline characteristics of all individuals and by their aMED score tertiles are presented in Table 1. Compared to individuals in the lowest aMED score tertile (T1), individuals in the highest tertile (T3) were more likely to be non-Hispanic White, highly educated, nulliparous, and had lower pre-pregnancy BMI and a healthier profile of dietary intake.

### 3.2. Prospective associations of aMED score reported in the first trimester

**Fig. 1.**
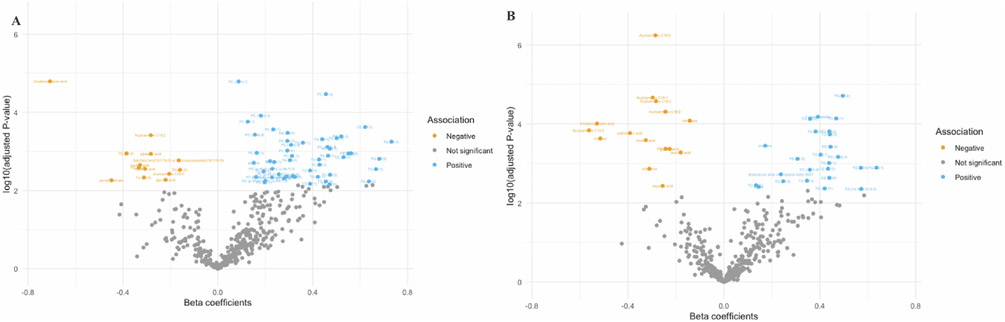
Volcano plots showing individual metabolite associated with peri-conception and first trimester alternate Mediterranean Diet (aMED) score reported at 8–13 weeks (A) and second trimester aMED score reported at 16–22 weeks (B). *β* coefficients were adjusted for age, race, education, pre-pregnancy BMI, and physical activity in multivariable linear regression analyses. Multiple comparisons were corrected using the Benjamini-Hochberg method with the false discovery rate (FDR) < 0.05 being considered as statistically significant.

Among 459 annotated metabolites, 64 were individually associated with aMED score reported in the first trimester (FDR<0.05), adjusting for age, race/ethnicity, education, pre-pregnancy BMI, and physical activity (Table 2). Among 64 metabolites, 51 (79.69%) had positive associations and 13 (20.31%) had negative associations (Fig. 1A and Supplementary Fig. 3A). In the sensitivity analysis, all 64 metabolites remained significantly associated with aMED score in individuals without GDM (Supplementary Table 1).

**Fig. 2.**
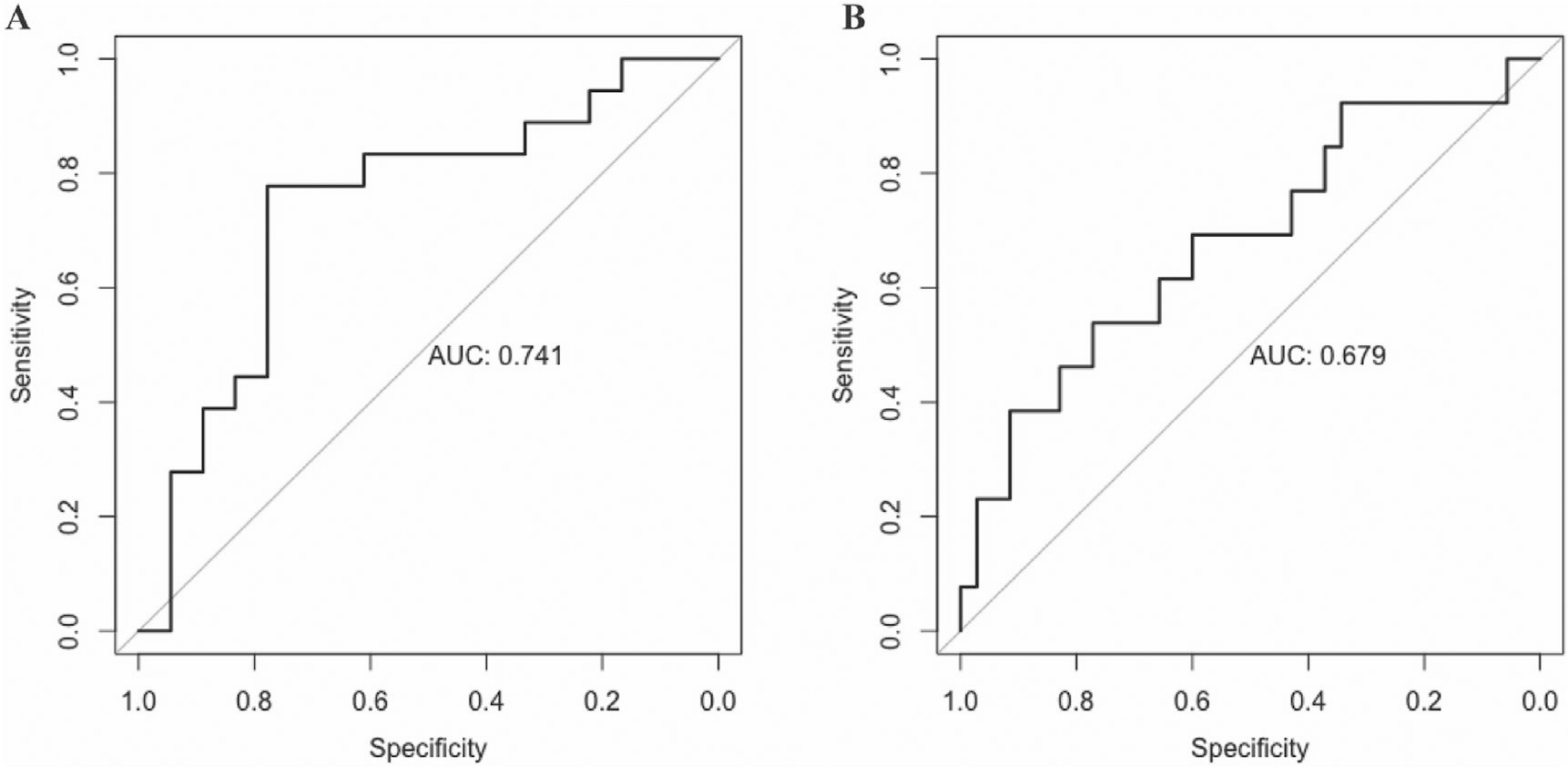
Receiver operating characteristic curves (ROCs) for panels of metabolites jointly associated with the peri-conception and first trimester alternate Mediterranean diet (aMED) score reported at 8–13 weeks (A) and the second trimester aMED score reported at 16–22 weeks (B) in LASSO regressions with 10-fold cross-validation. The 10-metabolite panel in the first trimester (i.e., CE (20:5) A, TG (49:1), TG (58:4), PC (33:1), PC (40:7), glutamic acid, aspartic acid, 3-hydroxybutyric acid, and epsilon-caprolactam) has the area under curve (AUC) of 0.74 (95% CI: 0.57, 0.91). The 3-metabolite panel in the second trimester (i.e., CE (20:5) B, acylcarnitine (C16:0), and glycolic acid) had an AUC of 0.68 (95% CI: 0.50, 0.86). Abbreviations: cholesterol ester (CE), triglycerides (TG), phosphatidylcholines (PC).

A panel of 10 metabolites was jointly associated with aMED score (AUC: 0.74; 95% CI: 0.57, 0.91) in LASSO regression, including positive associations of cholesterol ester (CE, 20:5) A, triglycerides (TG, 49:1), TG (58:4), phosphatidylcholines (PC, 33:1), and PC (40:7), and inverse associations of glutamic acid, aspartic acid, and 3-hydroxybutyric acid, and epsilon-caprolactam (Fig. 2). Except for the epsilon-caprolactam, all other 9 metabolites were also individually associated with aMED scores.

### 3.3. Cross–sectional associations of metabolites with aMED score reported in the second trimester

After adjusting for the abovementioned covariates, 41 metabolites were significantly associated (FDR< 0.05) with aMED score reported in the second trimester, with 25 (61.0%) having positive associations and 16 (39.0%) having negative associations (Fig. 1B and Supplementary Fig. 3B). Among the 41 metabolites, 14 (34%) were overlapped with the metabolites prospectively associated with aMED reported in the 1st trimester, including glycolic acid, aspartic acid, 3-hydroxybutyric acid, linoleic acid, acylcarnitine (C18:2) and (C18:0), TGs (58:4), TG (56:1) A and B, TG (60:2), PC (36:5) B, PC (42:6), and CE (20:5) A and B (Table 2). In the sensitivity analysis, all 41 metabolites remained significantly associated with aMED score in individuals without GDM (Supplementary Table 2).

A panel of 3 metabolites was jointly associated with aMED score with an AUC of 0.68 (95% CI: 0.50, 0.86) in the LASSO regression, including a positive association of CE (20:5) B and inverse associations of glycolic acid and acylcarnitine (C16:0). All 3 were also individually associated with the aMED score in linear regressions.

## 4. Discussions

In this longitudinal study among pregnant individuals, we identified several plasma metabolomic markers for the Mediterranean diet in peri-conception and 1st trimester or recent diet in the 2nd trimester. Most Mediterranean diet-related metabolites are lipids species (e.g., acylcarnitines, linoleic acid, long-chain TGs, PCs, and CEs); others are amino acids and derivatives, and sugar alcohols. Of note, 14 metabolites were significantly related to the Mediterranean diet in both time windows. In addition to individual metabolites, we also identified multi-metabolite panels for the Mediterranean diet in pregnant individuals at two time windows. Such multi-metabolite panels with good-to-excellent predictability are promising to be considered the potential biomarkers of the Mediterranean diet because they significantly reduced the numbers of metabolites (i.e., as compared with all individually associated metabolites) we need to measure in future applications.

We are unaware of previous studies investigating the metabolomic markers for the Mediterranean diet in pregnant individuals. In the current study among pregnant individuals, we replicated some Mediterranean-related metabolites which were reported in previous studies among non-pregnant populations, including several long-chain TGs and PCs, glyceryl palmitate, free fatty acids (palmitoleic acid, oleic acid, linoleic acid), medium/long-chain acylcarnitines (C16:0; C18:0; C18:1; C18:2), CE (20:5), and amino acids (aspartic acid, 3-hydroxybutyric acid, glycolic acid) [14-23]. We also identified novel markers such as glycolic acid, sugar alcohols (i.e., lyxitol and xylitol), and organic acids (i.e., citric acid and isocitric acid). Cross-population consistent findings are promising, supporting the concept that metabolomics could be a new approach for identifying biomarkers for dietary patterns across populations. The novel metabolites may reflect the variability of the physiological conditions of study populations (e.g., pregnant vs. non-pregnant), but could also be due to the differences in study design, population, metabolomic profiling, and statistical analysis approach.

Consistent with previous studies in non-pregnant populations [14-23], we found most metabolites associated with the Mediterranean diet among pregnant individuals are lipids species (i.e., CE (20:5) A and B, long-chain acylcarnitines (C18:0) and (C18:2), long-chain TGs (TG (56:1) A and B, TG (58:4), TG (60:2) A, and PC (36:5) B, and linoleic acid). As the esterified form of cholesterol with a single fatty acid, CE is the major form of cholesterol in human plasma [37]. The plasma levels of CEs have been linked with dietary intake of monosaturated fatty acids (MUFA) [38], olive oil, and seafood [21,39]. The health effects of individual CEs are unclear, but available evidence tends to suggest that CEs with longer, more unsaturated acyl chains may be favorable for cardiovascular diseases (CVD) [16] and diabetes [40]. Acylcarnitines have been identified as markers of meat (positive associations) [1,41,42], and coffee intakes [43] (inverse associations). Long-chain acylcarnitines were also positively associated with Western dietary patterns in a Canadian study [44]. As intermediates of fatty acid oxidation, disturbance of plasma acylcarnitines has been linked with the development of diabetes [45,46] and CVD [47,48] in the non-pregnant population, as well as, GDM and fetal development in pregnant individuals [49,50]. Indeed, findings from PREDIMED trial data have suggested that the Mediterranean diet may mitigate the adverse associations of acylcarnitines with CVD [16,51]. Plasma long-chain TGs and PCs mainly come from the consumption of fish, nuts, and vegetable oils (e.g. olive oil) [52]. Long-chain PCs have also been identified as markers of coffee consumption [53]. Plasma profiles of TGs and PC have been suggested as important signatures of insulin sensitivity and CVD risk [40,54]. TGs and PCs with different acyl chains and double bonds may play different roles, but the exact contribution of each or the combination of TGs and PCs is still poorly understood.

Linoleic acid was inversely associated with the Mediterranean diet at two-time windows in our study and a previous study in male Finnish smokers from the Alpha-Tocopherol, Beta-Carotene Cancer Prevention Study cohort [23]. As an essential fatty acid to humans, the only resource of linoleic acid is from dietary intake (primarily from vegetable oils, nuts, and seeds) [55]. In the human body, linoleic acid is the parental n-6 polyunsaturated fatty acid (PUFA) and can be converted to arachidonic acid and subsequently metabolized to pro-inflammatory lipids mediators (i.e., eicosanoids). On the other hand, α-linolenic acid, an *n*-3 PUFA, can be converted to eicosapentaenoic acid (EPA) and docosahexaenoic acid (DHA), which subsequently produce the anti-inflammatory lipid mediators (resolvins and protectins) [56]. Mediterranean diet has been linked with a well-balanced plasma linoleic/α-linolenic acid ratio, which may result in a lower risk of CVD through the inflammation responses [57,58].

Several amino acids and derivatives such as glutamic acid (a major excitatory neurotransmitter and a by-product of the branched-chain amino acids catabolism), aspartic acid (a metabolite involved in the urea cycle), and 3-hydroxybutyric acid (a by-product of short-chain fatty acids and branched-chain amino acids; also known as β-hydroxybutyric acid) were identified as metabolomic markers for Mediterranean diet in our study and two observations studies in non-pregnant populations [20,21]. These amino acid derivatives are mainly involved in the urea cycle during amino acid catabolism [59]. Previous studies have shown the association of glutamic and aspartic acids with central obesity [60-62], diabetic retinal disease [61,63], and risk of CVD [64]. More importantly, the glutamic acid/glutamate ratio has been identified as the single metabolite most strongly correlated with the visceral adipose tissue in several populations [62,65,66]. Taken together, it is possible that gluconeogenesis from amino acids could play important roles in the potential metabolic pathways that explain some health benefits of the Mediterranean diet.

We identified a couple of novel carbohydrate metabolites that were associated with the Mediterranean diet in pregnant individuals, including two sugar alcohols (i.e., lyxitol and xylitol), glycolic acid, and two organic acids (i.e., citric acid and isocitric acid). Xylitol and lyxitol are sugar alcohols that can be found naturally in many fruits and vegetables, or artificially produced [67]. As non-digestible carbohydrates, they have a sweet taste but produce much fewer calories in human bodies. Thus, they (mainly xylitol) are widely used as a sugar substitute in “sugar-free” products such as chewing gums, yogurt, cookies, and candies [67]. The plasma levels of these sugar alcohols are mainly from dietary sources through passive diffusion in the small intestine [68]. Therefore, the positive associations of xylitol and lyxitol with the Mediterranean diet may reflect the high consumption of fruit and vegetables, or some “commercially labeled “sugar-free” products, or both. Glycolic acid was inversely associated with the Mediterranean diet in both the first and second trimesters in our study of pregnant individuals, but not in previous studies in non-pregnant populations. Foods containing oxalic acid (e.g., spinach, rhubarb, and almond milk) or glyoxal (e.g. bread, cookies, yogurt, sardine oil, coffee, tea, beer, and wine) are likely the main exogenous sources of glycolic acid. However, most glycolic acids are produced endogenously from glycolaldehyde (a product of fructose and xylitol metabolism) during the synthesis of oxalate [69]. Clinically, glycolic acid is widely used in skin care products [70]. Urinary glycolic acid has also been inversely related to obesity and visceral fat tissue in healthy adults [71]. Citric acid and isocitric acid are tricarboxylic acid (TCA) cycle intermediates. TCA cycle is the final common oxidative pathway for carbohydrates, fats, and amino acids. It is the most important metabolic pathway for the energy supply in humans [72]. Citric acid is found in citrus fruits (e.g., oranges, lemons, and limes) and isocitric acid is rich in most berries (e.g., blackberries) and vegetables (e.g., carrots). Both of them are also commonly used as a flavoring (add sour taste) and preservative in food and beverages, especially soft drinks and candies. Urinary citric acid has been identified as the marker of dietary intake of wine and grape juice and lactovegetarian diet [73]. Serum isocitric acid has been linked to dementia in a small case–control study [74] and mild cognitive impairment in a large prospective study among Hispanic Community Health Study/Study of Latinos (HCHS/SOL) [75]. In pregnant individuals, first-trimester plasma citric acid has been identified as one of the top metabolites for predicting preeclampsia [76].

In the current study among pregnant individuals, we replicated some Mediterranean diet-related metabolites which were identified in previous studies among non-pregnant population. Among them, linoleic acid, aspartic acid, 3-hydroxybutyric acid, and CE (20:5) were associated with the Mediterranean diet in both time windows during the pregnancy, suggesting the robustness of the findings. The replicated findings are promising, supporting the concept that metabolomics could be a new approach for identifying biomarkers for dietary patterns across populations. The novel metabolites may reflect the variability of the physiological conditions of study populations (e.g., pregnant vs. non-pregnant), but could also be due to the differences in study design, population, metabolomic profiling, and statistical analysis approach.

Our study has several unique strengths. As the first study that investigated the metabolomic markers for Mediterranean diet in pregnant individuals, we recruited pregnant individuals from multiple racial and ethnic groups in 12 health centers in the US to enhance the generalizability of the study results to US pregnant women. We used longitudinal data with dietary intakes assessed prior to the plasma specimen collection to ensure the temporality of the association. Most previous studies only reported cross–sectional correlations [18-23,39]. Furthermore, we calculated the aMED score using a predefined method, which has been applied to both pregnant and non-pregnant populations, making our results to be easily compared with and replicated in other studies. In addition, we collected fasting blood samples that are less subjected to measurement variability and less influenced by other factors. As dietary patterns are combinations of foods and nutrients, a panel of metabolites could better capture the interrelations of nutrients and foods presented in dietary patterns with much smaller numbers of metabolites. In addition, with the availability of longitudinal dietary data before pregnancy and during early pregnancy, we were able to identify both long-term and short-term metabolite markers of Mediterranean diet. The multi-metabolite panels had fair-to-good predictability of adherence to Mediterranean diet in both first and second trimesters among pregnant individuals, suggesting the potential applications of using them as biomarkers of dietary intake.

Several limitations of this study should be considered when interpreting the results. First, our study is an observational study. Although we have examined and adjusted confounders rigorously, the residual confounding cannot be completely ruled out. Second, we only have one measure of 24-h recall at 2nd trimester, which may not be able to capture some foods that are usually consumed by study participants. Nevertheless, dietary assessments using one 24-h recall have been applied to derive the dietary patterns, including the Mediterranean diet, in both non-pregnant adult population [77] and pregnant individuals in large epidemiological studies [10]. Having meaningful numbers of overlapped metabolites from both the aMED scores derived from FFQ and one 24-h recall also suggested the usefulness of both dietary assessment methods. Future studies may need to quantify the levels of misclassification. Lastly, we only included the known metabolites, which could possibly miss some important metabolic features. However, these known metabolites are more reliable and can be used to compare with the results from other studies.

In conclusion, we detected a set of metabolites associated with the Mediterranean diet in pregnant individuals. Some of them were significantly related to the Mediterranean diet in both peri-conception and early-to-mid pregnancy time windows. These metabolites may deserve further investigations using the targeted approach to improve the accuracy of quantification and examining their relationships with pregnancy and fetal outcomes in future studies.

## Supplementary Material

Supplemental Material

## Figures and Tables

**Table 1:** Characteristics of pregnant individuals by tertiles of peri-conception and first trimester alternate Mediterranean Diet (aMED) score reported at 8–13 weeks, the NICHD Fetal Growth Studies–Singleton Cohort.

Characteristics^a^	All	aMED Score Tertile			
	N = 186	T1 (N = 65)	T2 (N = 80)	T3 (N = 41)	*P* ^b^
*aMED score, median (IQR)*	*4 (3–5)*	*2 (2–3)*	*5 (4–5)*	*7 (6–7)*	
Age, years	28.00 (0.40)	25.53 (0.67)	29.02 (0.58)	31.08 (0.61)	<0.001
Race/ethnicity, % (N)					
Non-Hispanic White	27.18 (37)	24.39 (13)	25.45 (17)	36.10 (7)	<0.001
Non-Hispanic Black	25.70 (23)	38.81 (13)	20.01 (7)	9.93 (3)	
Hispanic	24.39 (70)	25.63 (26)	25.32 (32)	20.10 (12)	
Asian/Pacific Islander	22.73 (56)	11.16 (13)	29.22 (24)	33.87 (19)	
Associate degree or more, N (%)	52.85 (102)	38.59 (27)	56.06 (47)	75.66 (28)	<0.001
Married/living with a partner, % (N)	70.14 (147)	63.34 (52)	73.18 (62)	78.19 (33)	<0.001
Nulliparous, % (N)	50.54 (81)	43.92 (26)	55.62 (35)	54.35 (20)	<0.001
Pre-pregnancy BMI, kg/m^2^	25.51 (0.38)	26.69 (0.72)	25.91 (0.60)	23.07 (0.48)	0.002
Pre-pregnancy BMI status, % (N)					
Normal (BMI 19.0-<25.0 kg/m^2^)	50.38 (84)	45.85 (24)	45.95 (34)	67.93 (26)	<0.001
Overweight (BMI: 25.0–29 0.9 kg/m^2^)	34.80 (63)	32.13 (24)	40.08 (28)	30.25 (11)	
Obese (BMI >30, kg/m^2^)	14.82 (39)	22.02 (17)	13.97 (18)	1.83 (4)	
Total physical activity, (MET-h)/week	307.61 (10.59)	307.43 (19.67)	323.44 (15.74)	278.11 (18.41)	0.30
Dietary intakes^c^					
Total energy, kcal/day	2191 (71.19)	1871 (96.61)	2419 (125.96)	2408 (130.14)	<0.001
Total carbohydrate, g/day	299.6 (10.82)	252.6 (12.84)	335.3 (20.83)	322.5 (18.00)	0.001
Total protein, g/day	86.15 (2.98)	71.51 (4.04)	91.65 (4.79)	105.40 (6.46)	<0.001
Total fatty acids, g/day	77.71 (2.81)	67.76 (4.45)	84.95 (4.57)	84.17 (5.40)	0.01
Saturated fatty acids, g/day	25.51 (1.03)	23.99 (1.67)	27.30 (1.78)	25.21 (1.77)	0.36
Monounsaturated fatty acids, g/day	29.65 (1.13)	24.83 (1.74)	32.65 (1.80)	33.73 (2.22)	0.001
Polyunsaturated fatty acids, g/day	16.40 (0.60)	13.62 (0.81)	18.40 (0.88)	18.24 (1.21)	<0.001
Fiber, g/day	22.37 (0.84)	16.62 (0.88)	24.15 (1.33)	30.65 (1.89)	<0.001
Vegetables, serving/day	3.66 (0.19)	2.44 (0.26)	4.23 (0.30)	5.03 (0.40)	<0.001
Fruit, serving/day	6.39 (0.45)	4.44 (0.45)	7.60 (0.92)	8.04 (0.71)	0.001
Whole grains, g/day	27.65 (1.42)	24.80 (2.58)	25.22 (1.75)	37.99 (3.13)	0.001
Nuts and legumes, serving/day	0.67 (0.05)	0.29 (0.04)	0.78 (0.07)	1.24 (0.13)	<0.001
Red and processed meats, serving/day	0.47 (0.03)	0.45 (0.05)	0.53 (0.05)	0.41 (0.05)	0.28
Fish, serving/day	0.31 (0.03)	0.17 (0.02)	0.29 (0.04)	0.62 (0.09)	<0.001

Abbreviations: aMED - alternate Mediterranean Diet; NICHD – *Eunice Kennedy Shriver* National Institute of Child Health and Human Development; BMI: body mass index; MET-h: metabolic equivalent hours.

a Data are presented as weighted percentage and unweighted frequency % (N) for categorical variables and weighted mean (standard errors, SE) for continuous variables. Sampling weights were applied to all analyses to represent the full NICHD Fetal Growth Studies–Singletons population.

b P-values were compared across the tertiles dietary score using one-way ANOVA for continuous variables and χ^b^-tests for categorical variables.

c Dietary intakes in the past 3 months (i.e., the habitual diet during the peri-conception and 1st trimester) at 8–13 weeks (visit 0) using a semi-quantitative Food Frequency Questionnaire (FFQ).

**Table 2:** Metabolites associated with peri-conception and first trimester alternate Mediterranean Diet (aMED) score reported at 8–13 weeks and second trimester aMED score reported 16–22 weeks, the NICHD Fetal Growth Studies–Singleton Cohort.

Superclasses^b^	Classes^b^	Metabolites^a^	8–13 weeks		16–22 weeks	
			Coefficients	FDRs^c^	Coefficients	FDRs^c^
Lipids and lipid-like molecules	Acylcarnitine	Acylcarnitine (C10:0)			−0.56	0.01
		Acylcarnitine (C12:0)			−0.86	<0.001
		Acylcarnitine (C16:0)			−0.29	<0.001
		Acylcarnitine (C18:0)	−0.20	0.04	−0.24	0.004
		Acylcarnitine (C18:1)			−0.30	0.002
		Acylcarnitine (C18:2)	−0.28	0.02	−0.28	0.002
	Sterol lipids/Cholesterol esters (CE)	CE (20:5) A	0.62	0.02	0.44	0.03
		CE (20:5) B	0.53	0.02	0.43	0.02
	Fatty Acyls/SFA	Lauric acid (C12:0)			0.24	0.03
	Fatty Acyls/MUFA	Palmitoleic acid (C16:1)			−0.39	0.01
		Oleic acid (C18:1)			−0.31	0.02
	Fatty Acyls/PUFA	Linoleic acid (C18:2)	−0.33	0.03	−0.33	0.01
	Glycerolipids/Monoacylglycerols	1-monopalmitin			0.39	0.00
		(glyceryl palmitate)				
	Glycerolipids/Diacylglycerols	DG (38:5) A	0.29	0.02		
		DG (38:5) B	0.29	0.02		
	Glycerolipids/Triacylglycerols	TG (14:0/14:0/14:0)			0.57	0.05
		TG (44:1) A	0.79	0.03		
		TG (44:1) B	0.67	0.03		
		TG (46:0)	0.68	0.02		
		TG (46:1) A	0.56	0.02		
		TG (46:1) B	0.73	0.02		
		TG (46:2)	0.64	0.04		
		TG (48:0)	0.47	0.05		
		TG (48:1) A	0.43	0.02		
		TG (48:1) B	0.44	0.02		
		TG (48:2) A	0.30	0.02		
		TG (48:2) B	0.43	0.03		
		TG (49:0)	0.47	0.04		
		TG (49:1) A	0.36	0.02		
		TG (49:1) B	0.39	0.05		
		TG (50:1)	0.19	0.03		
		TG (53:5)	−0.31	0.04		
		TG (54:3)	−0.16	0.03		
		TG (54:5) A	−0.33	0.03		
		TG (54:5) B	0.45	0.04		
		TG (54:6)	−0.39	0.02		
		TG (54:8) A			0.57	0.02
		TG (54:8) B			0.35	0.04
		TG (56:1) A	0.42	0.04	0.47	0.00
		TG (56:1) B	0.46	0.02	0.40	0.01
		TG (56:2)			0.44	0.01
		TG (58:1) A			0.44	0.02
		TG (58:1) B			0.64	0.02
		TG (58:2) A			0.50	0.00
		TG (58:2) B			0.38	0.01
		TG (58:3)			0.31	0.01
		TG (58:4)	0.50	0.02	0.13	0.04
		TG (58:9)			0.25	0.04
		TG (60:2) A	0.32	0.04	0.36	0.00
		TG (60:2) B			0.44	0.01
		TG (60:11)			0.42	0.05
	Glycerophospholipids/phosphatidylcholine	PC (28:0)	0.55	0.02		
		PC (30:0)	0.29	0.04		
		PC (30:1)	0.47	0.02		
		PC (32:1)	0.31	0.02		
		PC (33:1)	0.24	0.03		
		PC (34:1)	0.13	0.02		
		PC (34:3)			−0.24	0.01
		PC (34:4)	0.28	0.04		
		PC (35:1)	0.20	0.05		
		PC (36:1)	0.16	0.02		
		PC (36:4) C	0.09	0.004		
		PC (36:5) B	0.46	0.02	0.36	0.02
		PC (36:6)	0.46	0.01		
		PC (37:6)	0.32	0.04		
		PC (38:4) B	0.16	0.02		
		PC (38:5) A	0.16	0.04		
		PC (38:6) A	0.18	0.01		
		PC (39:6)	0.29	0.02		
		PC (40:6) A			0.48	0.01
		PC (40:6) B	0.31	0.02		
		PC (p-40:6) or PC (o-40:7) A	0.23	0.03		
		PC (p-40:6) or PC (o-40:7) B	0.20	0.04		
		PC (40:7)	0.23	0.02		
		PC (42:6)	0.52	0.02		
		PC (42:6)			0.44	0.01
		PC (p-44:5) or PC (o-44:6)	0.26	0.04		
	Sphingolipids/glucosyl	Lactosylceramide (d18:1/16:0)	−0.16	0.02		
Organic acids and derivatives	Amino acids and derivatives	Alanine	0.15	0.03		
		Aspartic acid	−0.31	0.03	−0.26	0.04
		Glutamic acid	−0.28	0.02		
		3-hydroxybutyric acid	−0.71	0.004	−0.53	0.00
		Ornithine			−0.14	0.00
	Alpha-hydroxy acid (AHA)	Glycolic acid	−0.22	0.04	−0.23	0.01
	Quinolones and derivatives	Hydroxycarbamate	0.23	0.04		
	Tricarboxylic acids and derivatives	Citric acid			−0.14	0.00
		Isocitric acid			−0.18	0.01
Organic oxygen compounds	Sugar alcohol	Lyxitol	0.22	0.02	0.17	0.01
		Xylitol			0.15	0.05
	Sugar/Disaccharide	Maltose			−0.52	0.01
Homogeneous non-metal compounds	Non-metal oxoanionic compounds	Pyrophosphate	−0.45	0.04		

Abbreviations: CE, cholesteryl esters; DG, diglyceride; GW, gestational week; LPC, lysophosphatidylcholine; NICHD, *Eunice Kennedy Shriver* National Institute of Child Health and Human Development; PC, phosphatidylcholine; SM, sphingomyelin; TG, triglyceride.

a Metabolites that were individually and significantly associated with the alternate Mediterranean Diet (aMED) scores in multivariable linear regression analyses after adjusting for age, race, education, pre-pregnancy BMI, and physical activity.

b Classification of chemical compound classes was performed using ClassyFire.

c Multiple comparisons were corrected using the Benjamini-Hochberg method with the false discovery rate (FDR) < 0.05 being considered as statistically significant.

## Data Availability

Data described in the manuscript, code book, and analytic code will be available upon request pending application and approval of a data-sharing agreement.
