# Supplementary material for: Metabolomic biomarkers of the mediterranean diet in pregnant individuals: A prospective study
Source: Clin Nutr. Author manuscript; Available in PMC 2023 Mar 21. (PMC10029322; doi:10.1016/j.clnu.2023.01.011)
Supplement: Supplemental Material [file NIHMS1876026-supplement-Supplemental_Material.docx]

**Supplementary Materials**

**Title:** **Metabolomic biomarkers of the Mediterranean diet in pregnant individuals**

**Authors:** Liwei Chen, Jin Dai, Zhe Fei, Xinyue Liu, Yeyi Zhu, Mohammad L. Rahman, Ruijin Lu, Susanna D. Mitro, Jiaxi Yang, Stefanie N. Hinkle, Zhen Chen, Yiqing Song, Cuilin Zhang.

**Supplementary Table 1**. Associations between 64 metabolites and aMED score reported at 8-13 weeks of gestation by gestational diabetes mellitus^1^

|  | **All (*n*=186)** | | | **Non-GDM (*n*=117)** | | | **GDM (*n*=68)** | | |
| --- | --- | --- | --- | --- | --- | --- | --- | --- | --- |
| **Metabolite** | **Coefficient** | **P** | **Q** | **Coefficient** | **P** | **Q** | **Coefficient** | **P** | **Q** |
| 3-hydroxybutyric acid | -0.71 | <0.001 | 0.004 | -0.77 | <0.001 | 0.01 | 0.73 | 0.01 | 0.19 |
| PC (36:4) C | 0.09 | <0.001 | 0.004 | 0.09 | <0.001 | 0.01 | -0.02 | 0.69 | 0.92 |
| PC (36:6) | 0.46 | <0.001 | 0.01 | 0.47 | 0.001 | 0.02 | 0.14 | 0.38 | 0.65 |
| PC (38:6) A | 0.18 | <0.001 | 0.01 | 0.19 | 0.002 | 0.02 | 0.10 | 0.13 | 0.49 |
| PC (34:1) | 0.13 | <0.001 | 0.02 | 0.13 | 0.002 | 0.02 | 0.00 | 0.99 | 0.99 |
| Acylcarnitine C18:2 | -0.28 | <0.001 | 0.02 | -0.32 | 0.002 | 0.02 | 0.37 | 0.02 | 0.33 |
| TG (48:1) | 0.44 | <0.001 | 0.02 | 0.48 | 0.003 | 0.02 | -0.26 | 0.21 | 0.54 |
| TG (49:1) | 0.36 | <0.001 | 0.02 | 0.39 | 0.004 | 0.02 | -0.16 | 0.34 | 0.63 |
| TG (58:4) | 0.50 | <0.001 | 0.02 | 0.51 | 0.005 | 0.02 | 0.39 | 0.22 | 0.54 |
| PC (40:7) | 0.23 | <0.001 | 0.02 | 0.23 | 0.005 | 0.02 | 0.16 | 0.07 | 0.46 |
| PC (42:6) | 0.52 | <0.001 | 0.02 | 0.54 | 0.004 | 0.02 | -0.04 | 0.88 | 0.99 |
| PC (38:4) B | 0.16 | <0.001 | 0.02 | 0.16 | 0.003 | 0.02 | 0.07 | 0.51 | 0.74 |
| DG (38:5) | 0.29 | 0.001 | 0.02 | 0.30 | 0.001 | 0.02 | 0.10 | 0.57 | 0.82 |
| DG (38:5) | 0.29 | 0.000 | 0.02 | 0.30 | 0.002 | 0.02 | 0.07 | 0.70 | 0.92 |
| CE (20:5) | 0.62 | 0.000 | 0.02 | 0.65 | 0.002 | 0.02 | -0.01 | 0.97 | 0.99 |
| TG (46:1) | 0.73 | 0.001 | 0.02 | 0.79 | 0.002 | 0.02 | -0.41 | 0.21 | 0.54 |
| PC (40:6) B | 0.31 | 0.001 | 0.02 | 0.32 | 0.01 | 0.02 | 0.23 | 0.03 | 0.33 |
| PC (36:5) B | 0.46 | 0.001 | 0.02 | 0.47 | 0.01 | 0.02 | 0.20 | 0.36 | 0.64 |
| PC (30:1) | 0.47 | 0.001 | 0.02 | 0.51 | 0.01 | 0.02 | -0.22 | 0.40 | 0.67 |
| Glutamic acid | -0.28 | 0.001 | 0.02 | -0.29 | 0.01 | 0.02 | 0.00 | 0.99 | 0.99 |
| PC (28:0) | 0.55 | 0.001 | 0.02 | 0.59 | 0.01 | 0.02 | -0.24 | 0.31 | 0.63 |
| PC (39:6) | 0.29 | 0.001 | 0.02 | 0.30 | 0.01 | 0.02 | 0.24 | 0.07 | 0.46 |
| PC (36:1) | 0.16 | 0.001 | 0.02 | 0.17 | 0.01 | 0.02 | 0.09 | 0.44 | 0.71 |
| TG (54:6) | -0.39 | 0.001 | 0.02 | -0.42 | 0.01 | 0.02 | 0.27 | 0.20 | 0.54 |
| TG (46:1) | 0.56 | 0.001 | 0.02 | 0.61 | 0.01 | 0.02 | -0.36 | 0.13 | 0.49 |
| TG (56:1) | 0.46 | 0.001 | 0.02 | 0.49 | 0.01 | 0.02 | -0.02 | 0.93 | 0.99 |
| PC (32:1) | 0.31 | 0.001 | 0.02 | 0.34 | 0.01 | 0.02 | -0.15 | 0.44 | 0.71 |
| CE (20:5) | 0.53 | 0.001 | 0.02 | 0.55 | 0.01 | 0.02 | 0.13 | 0.60 | 0.83 |
| TG (46:0) | 0.68 | 0.002 | 0.02 | 0.75 | 0.01 | 0.02 | -0.55 | 0.12 | 0.49 |
| TG (48:1) | 0.43 | 0.002 | 0.02 | 0.46 | 0.01 | 0.02 | -0.23 | 0.33 | 0.63 |
| Lactosylceramide (d18:1/16:0) | -0.16 | 0.002 | 0.02 | -0.16 | 0.01 | 0.02 | -0.19 | 0.03 | 0.33 |
| TG (48:2) | 0.30 | 0.002 | 0.02 | 0.33 | 0.01 | 0.02 | -0.14 | 0.33 | 0.63 |
| Lyxitol | 0.22 | 0.002 | 0.02 | 0.23 | 0.01 | 0.02 | 0.01 | 0.92 | 0.99 |
| PC (33:1) | 0.24 | 0.002 | 0.03 | 0.25 | 0.01 | 0.02 | 0.02 | 0.88 | 0.99 |
| Alanine | 0.15 | 0.002 | 0.03 | 0.16 | 0.01 | 0.02 | -0.10 | 0.13 | 0.49 |
| Linoleic acid | -0.33 | 0.002 | 0.03 | -0.37 | 0.01 | 0.02 | 0.57 | 0.00 | 0.19 |
| TG (48:2) | 0.43 | 0.002 | 0.03 | 0.46 | 0.01 | 0.02 | -0.17 | 0.48 | 0.74 |
| TG (44:1) | 0.79 | 0.002 | 0.03 | 0.85 | 0.01 | 0.02 | -0.46 | 0.18 | 0.52 |
| TG (54:5) | -0.33 | 0.003 | 0.03 | -0.36 | 0.01 | 0.02 | 0.08 | 0.79 | 0.99 |
| Aspartic acid | -0.31 | 0.003 | 0.03 | -0.30 | 0.02 | 0.03 | -0.26 | 0.17 | 0.52 |
| PC (p-40:6) or PC (o-40:7) A | 0.23 | 0.003 | 0.03 | 0.24 | 0.02 | 0.02 | 0.14 | 0.23 | 0.55 |
| TG (44:1) | 0.67 | 0.003 | 0.03 | 0.72 | 0.01 | 0.02 | -0.46 | 0.11 | 0.49 |
| TG (54:3) | -0.16 | 0.003 | 0.03 | -0.18 | 0.01 | 0.02 | 0.28 | 0.01 | 0.19 |
| TG (49:0) | 0.39 | 0.003 | 0.03 | 0.42 | 0.01 | 0.02 | -0.24 | 0.31 | 0.63 |
| TG (50:1) | 0.19 | 0.003 | 0.03 | 0.21 | 0.01 | 0.02 | -0.15 | 0.15 | 0.49 |
| Acylcarnitine C18:0 | -0.20 | 0.004 | 0.04 | -0.23 | 0.01 | 0.02 | 0.38 | 0.12 | 0.49 |
| PC (p-44:5) or PC (o-44:6) | 0.26 | 0.004 | 0.04 | 0.27 | 0.02 | 0.02 | 0.04 | 0.79 | 0.99 |
| TG (49:0) | 0.47 | 0.004 | 0.04 | 0.51 | 0.01 | 0.02 | -0.20 | 0.50 | 0.74 |
| TG (56:1) | 0.42 | 0.004 | 0.04 | 0.44 | 0.02 | 0.02 | -0.06 | 0.81 | 0.99 |
| TG (60:2) | 0.32 | 0.005 | 0.04 | 0.34 | 0.02 | 0.03 | 0.04 | 0.84 | 0.99 |
| PC (37:6) | 0.32 | 0.004 | 0.04 | 0.32 | 0.03 | 0.03 | 0.23 | 0.18 | 0.52 |
| PC (30:0) | 0.29 | 0.004 | 0.04 | 0.32 | 0.02 | 0.02 | -0.29 | 0.08 | 0.49 |
| PC (38:5) A | 0.16 | 0.004 | 0.04 | 0.16 | 0.03 | 0.03 | -0.01 | 0.95 | 0.99 |
| TG (53:5) | -0.31 | 0.005 | 0.04 | -0.34 | 0.01 | 0.02 | 0.34 | 0.05 | 0.42 |
| PC (34:4) | 0.28 | 0.005 | 0.04 | 0.29 | 0.02 | 0.03 | -0.13 | 0.48 | 0.74 |
| Glycolic acid | -0.22 | 0.01 | 0.04 | -0.24 | 0.02 | 0.02 | 0.08 | 0.61 | 0.83 |
| Pyrophosphate | -0.45 | 0.01 | 0.04 | -0.45 | 0.03 | 0.03 | -0.31 | 0.34 | 0.63 |
| PC (p-40:6) or PC (o-40:7) B | 0.20 | 0.01 | 0.04 | 0.21 | 0.02 | 0.03 | -0.01 | 0.97 | 0.99 |
| TG (54:5) | 0.45 | 0.01 | 0.04 | 0.40 | 0.05 | 0.05 | 0.79 | 0.05 | 0.43 |
| TG (46:2) | 0.64 | 0.01 | 0.04 | 0.68 | 0.02 | 0.03 | -0.30 | 0.34 | 0.63 |
| PC (35:1) | 0.20 | 0.01 | 0.05 | 0.20 | 0.03 | 0.03 | 0.16 | 0.28 | 0.63 |
| TG (49:1) | 0.39 | 0.01 | 0.05 | 0.41 | 0.02 | 0.03 | -0.03 | 0.92 | 0.99 |
| TG (48:0) | 0.47 | 0.01 | 0.05 | 0.52 | 0.02 | 0.03 | -0.44 | 0.14 | 0.49 |

^1^ Multivariable linear regression analysis was performed adjusted for age, race, education, pre-pregnancy BMI, and physical activity. Multiple comparisons were corrected using the Benjamini-Hochberg method with the false discovery rate (FDR) <0.05 being considered as statistically significant.

Abbreviations: BMI, body mass index; CE, cholesteryl ester; DG, diacylglycerol; PC, phosphatidylcholine; TG, triacylglycerol.

**Supplementary Table 2**. Associations between 41 metabolites and aMED score reported at 16-22 weeks of gestation by gestational diabetes mellitus^1^

|  | **All (*n*=181)** | | | **Non-GDM (*n*=114)** | | | **GDM (*n*=66)** | | |
| --- | --- | --- | --- | --- | --- | --- | --- | --- | --- |
| **Metabolite** | **Coefficient** | **P** | **Q** | **Coefficient** | **P** | **Q** | **Coefficient** | **P** | **Q** |
| Acylcarnitine C12:0 | -0.86 | <0.001 | <0.001 | -0.91 | <0.001 | 0.001 | -0.20 | 0.50 | 0.90 |
| Acylcarnitine C16:0 | -0.29 | <0.001 | <0.001 | -0.30 | <0.001 | 0.001 | -0.03 | 0.76 | 0.90 |
| Acylcarnitine C18:2 | -0.28 | <0.001 | 0.002 | -0.30 | 0.001 | 0.004 | -0.10 | 0.48 | 0.90 |
| Acylcarnitine C18:1 | -0.30 | <0.001 | 0.002 | -0.32 | <0.001 | 0.004 | 0.04 | 0.77 | 0.90 |
| TG (58:2) | 0.50 | <0.001 | 0.002 | 0.51 | 0.001 | 0.004 | 0.44 | 0.04 | 0.60 |
| Acylcarnitine C18:0 | -0.24 | <0.001 | 0.004 | -0.25 | <0.001 | 0.004 | -0.15 | 0.47 | 0.90 |
| 1-monopalmitin | 0.39 | <0.001 | 0.004 | 0.42 | 0.001 | 0.004 | -0.22 | 0.17 | 0.90 |
| TG (56:1) | 0.47 | <0.001 | 0.004 | 0.49 | 0.001 | 0.01 | 0.26 | 0.20 | 0.90 |
| TG (60:2) | 0.36 | <0.001 | 0.004 | 0.37 | 0.002 | 0.01 | 0.33 | 0.02 | 0.45 |
| citric acid | -0.14 | <0.001 | 0.004 | -0.15 | 0.001 | 0.01 | -0.06 | 0.36 | 0.90 |
| 3-hydroxybutyric acid | -0.53 | <0.001 | 0.004 | -0.56 | 0.001 | 0.01 | -0.21 | 0.38 | 0.90 |
| Acylcarnitine C10:0 | -0.56 | <0.001 | 0.01 | -0.61 | 0.001 | 0.01 | 0.01 | 0.98 | 0.98 |
| TG (58:2) | 0.38 | <0.001 | 0.01 | 0.39 | 0.002 | 0.01 | 0.28 | 0.07 | 0.61 |
| TG (60:2) | 0.44 | <0.001 | 0.01 | 0.45 | 0.003 | 0.01 | 0.42 | 0.02 | 0.45 |
| Palmitoleic acid | -0.39 | <0.001 | 0.01 | -0.41 | 0.002 | 0.01 | -0.15 | 0.39 | 0.90 |
| TG (56:2) | 0.44 | <0.001 | 0.01 | 0.46 | 0.002 | 0.01 | 0.09 | 0.74 | 0.90 |
| Maltose | -0.52 | <0.001 | 0.01 | -0.53 | 0.003 | 0.01 | 0.08 | 0.73 | 0.90 |
| Linoleic acid | -0.33 | <0.001 | 0.01 | -0.34 | 0.003 | 0.01 | -0.04 | 0.80 | 0.90 |
| Ornithine | 0.17 | <0.001 | 0.01 | 0.18 | 0.004 | 0.01 | 0.07 | 0.48 | 0.90 |
| PC (42:6) | 0.44 | <0.001 | 0.01 | 0.48 | 0.003 | 0.01 | -0.21 | 0.37 | 0.90 |
| Glycolic acid | -0.23 | <0.001 | 0.01 | -0.24 | 0.003 | 0.01 | 0.15 | 0.24 | 0.90 |
| PC (34:3) | -0.24 | <0.001 | 0.01 | -0.25 | 0.004 | 0.01 | -0.03 | 0.81 | 0.90 |
| Isocitric acid | -0.18 | 0.001 | 0.01 | -0.19 | 0.003 | 0.01 | 0.06 | 0.56 | 0.90 |
| TG (56:1) | 0.40 | 0.001 | 0.01 | 0.43 | 0.004 | 0.01 | 0.09 | 0.54 | 0.90 |
| PC (40:6) A | 0.48 | 0.001 | 0.01 | 0.50 | 0.01 | 0.01 | 0.20 | 0.26 | 0.90 |
| TG (58:3) | 0.31 | 0.001 | 0.01 | 0.32 | 0.01 | 0.01 | 0.20 | 0.19 | 0.90 |
| TG (58:1) | 0.44 | 0.001 | 0.02 | 0.45 | 0.01 | 0.01 | 0.47 | 0.09 | 0.61 |
| Oleic acid | -0.31 | 0.001 | 0.02 | -0.33 | 0.01 | 0.01 | 0.09 | 0.60 | 0.90 |
| TG (58:1) | 0.64 | 0.001 | 0.02 | 0.69 | 0.01 | 0.01 | -0.07 | 0.80 | 0.90 |
| TG (54:8) | 0.57 | 0.001 | 0.02 | 0.61 | 0.01 | 0.01 | 0.14 | 0.67 | 0.90 |
| CE (20:5) | 0.43 | 0.001 | 0.02 | 0.46 | 0.01 | 0.01 | -0.12 | 0.58 | 0.90 |
| PC (36:5) B | 0.36 | 0.001 | 0.02 | 0.38 | 0.01 | 0.01 | -0.14 | 0.46 | 0.90 |
| CE (20:5) | 0.44 | 0.002 | 0.03 | 0.46 | 0.01 | 0.01 | 0.03 | 0.93 | 0.97 |
| TG (58:4) | 0.35 | 0.003 | 0.04 | 0.37 | 0.01 | 0.01 | 0.08 | 0.76 | 0.90 |
| TG (58:9) | 0.25 | 0.003 | 0.04 | 0.27 | 0.01 | 0.01 | -0.08 | 0.62 | 0.90 |
| TG (54:5) | 0.13 | 0.004 | 0.04 | 0.15 | 0.01 | 0.01 | -0.16 | 0.09 | 0.61 |
| Aspartic acid | -0.26 | 0.004 | 0.04 | -0.27 | 0.02 | 0.02 | 0.16 | 0.35 | 0.90 |
| Xylitol | 0.15 | 0.004 | 0.05 | 0.15 | 0.02 | 0.02 | -0.01 | 0.88 | 0.95 |
| TG (60:11) | 0.42 | 0.004 | 0.05 | 0.44 | 0.02 | 0.02 | 0.06 | 0.81 | 0.90 |
| TG (14:0/14:0/14:0) | 0.57 | 0.004 | 0.05 | 0.62 | 0.02 | 0.02 | -0.12 | 0.65 | 0.90 |

^1^ Multivariable linear regression analysis was performed adjusted for age, race, education, pre-pregnancy BMI, and physical activity. Multiple comparisons were corrected using the Benjamini-Hochberg method with the false discovery rate (FDR) <0.05 being considered as statistically significant.

Abbreviations: BMI, body mass index; CE, cholesteryl ester; PC, phosphatidylcholine; TG, triacylglycerol.

**Supplementary Figure 1.** Flow chart of the selection of the analytical population within the NICHD Fetal Growth Studies-Singleton Cohort


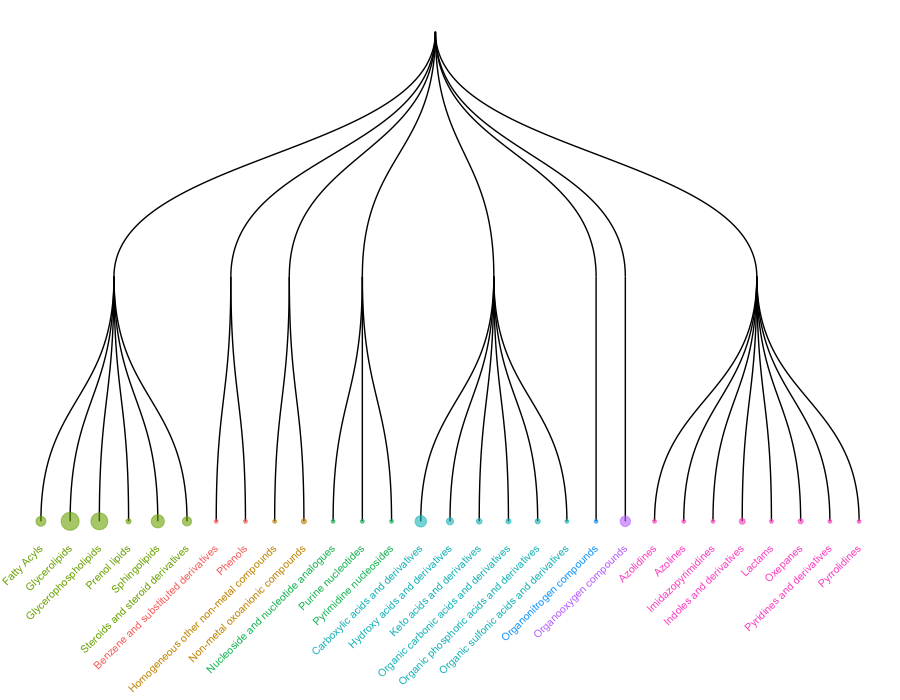


**Supplementary Figure 2**. Dendrogram of the analyzed metabolites by superclass and class, for which the classification of the chemical compound was performed using ClassyFire. From left to right, the superclasses are lipids and lipid-like molecules (in lime color, n=353), benzenoids (in coral color, n=2), homogeneous non-metal compounds (in pumpkin color, n=3), nucleosides, nucleotides, and analogues (in olive color, n=3), organic acids and derivatives (in hunter color, n=55), organic nitrogen compounds (in sea foam color, n=1), organic oxygen compounds (in periwinkle color, n=30), organoheterocyclic compounds (in plum color, n=12), respectively. The node size is related to the number of metabolites in each class.

A B


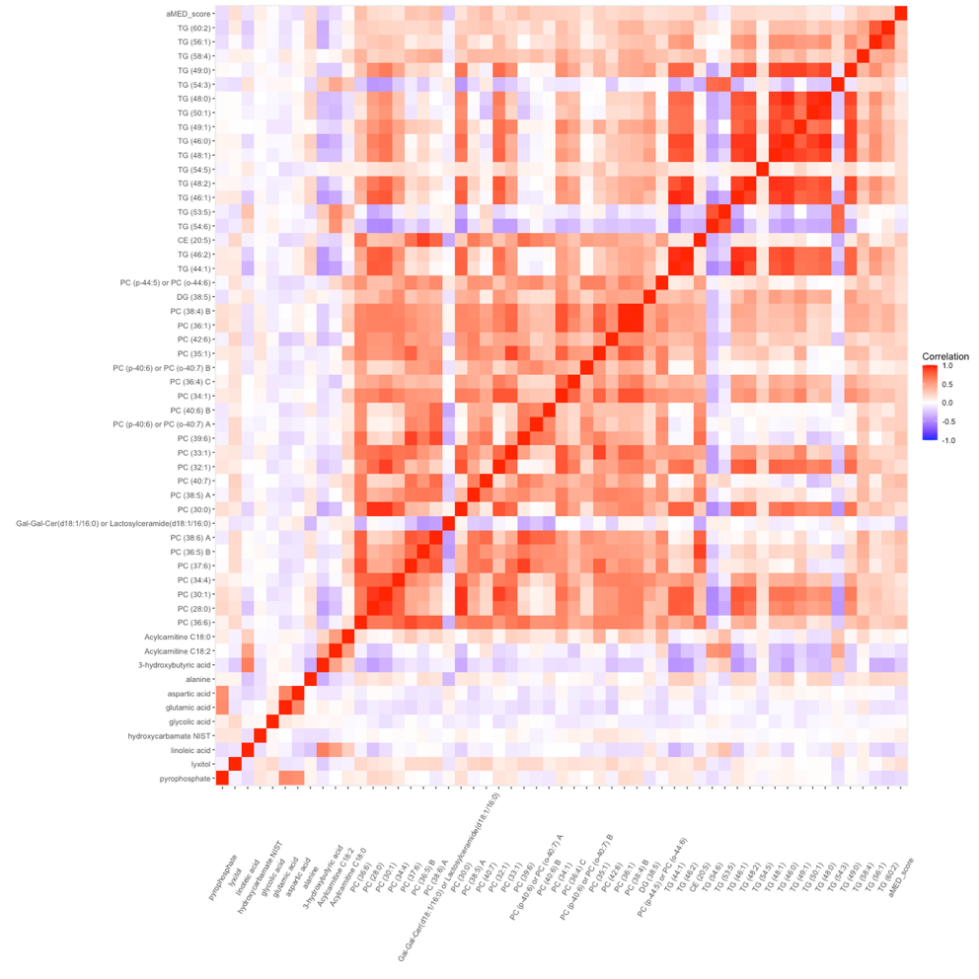

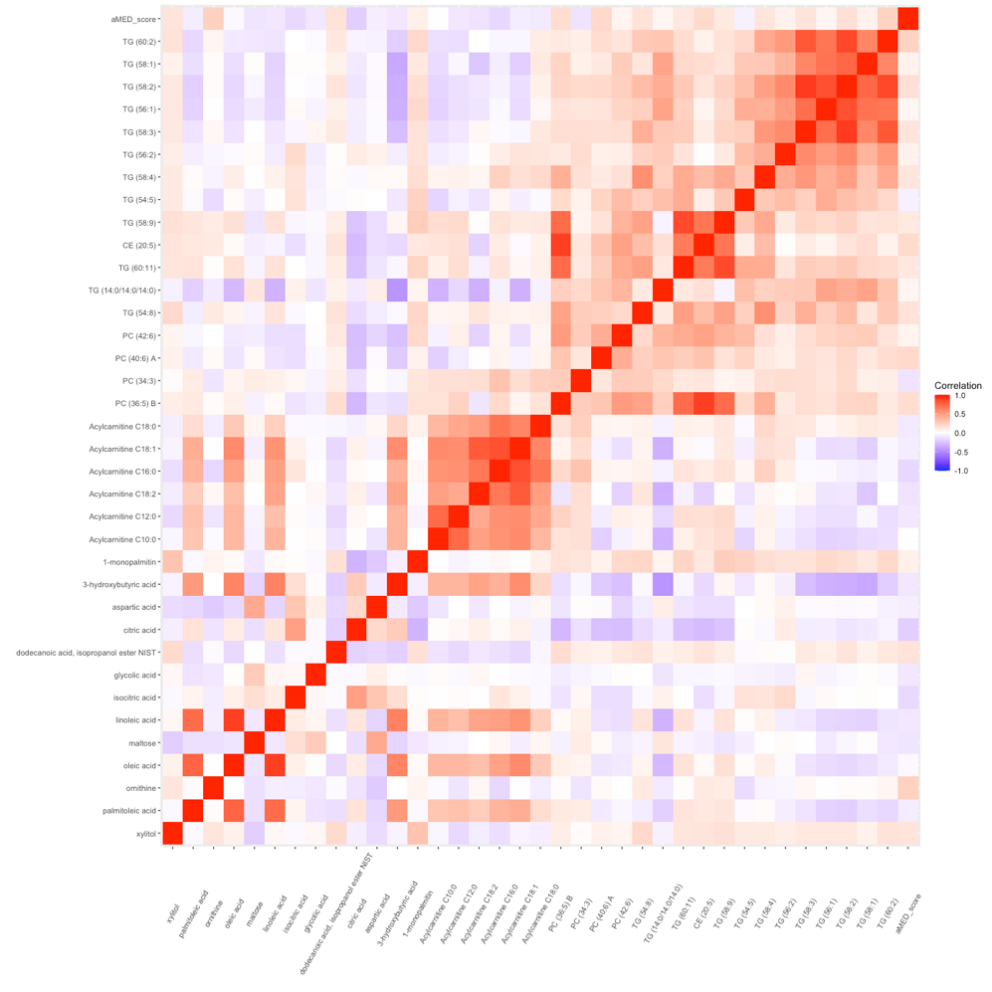


**Supplement Figure 3**. Heatmap of the correlation matrix of metabolites significantly associated with the aMED score at 8-13 weeks of gestation (A) and at 16-22 weeks of gestation (B). The correlation matrix was obtained using the Spearman correlation coefficients. Red represents a positive correlation and blue represents a negative correlation.
